# GraphBio: A shiny web app to easily perform popular visualization analysis for omics data

**DOI:** 10.3389/fgene.2022.957317

**Published:** 2022-09-07

**Authors:** Tianxin Zhao, Zelin Wang

**Affiliations:** ^1^ Department of Pediatric Urology, Guangzhou Women and Children’s Medical Center, Guangzhou Medical University, Guangdong Provincial Clinical Research Center for Child Health, Guangzhou, Guangdong, China; ^2^ Department of Bioinformatics, Shuzhi Biotech, LLC, Guangzhou, Guangdong, China

**Keywords:** omics, heatmap, volcano plots, visualization, GraphBio

## Abstract

**Background:** Massive amounts of omics data are produced and usually require sophisticated visualization analysis. These analyses often require programming skills, which are difficult for experimental biologists. Thus, more user-friendly tools are urgently needed.

**Methods and Results:** Herein, we present GraphBio, a shiny web app to easily perform visualization analysis for omics data. GraphBio provides 15 popular visualization analysis methods, including heatmap, volcano plots, MA plots, network plots, dot plots, chord plots, pie plots, four quadrant diagrams, Venn diagrams, cumulative distribution curves, principal component analysis (PCA), survival analysis, receiver operating characteristic (ROC) analysis, correlation analysis, and text cluster analysis. It enables experimental biologists without programming skills to easily perform popular visualization analysis and get publication-ready figures.

**Conclusion:** GraphBio, as an online web application, is freely available at http://www.graphbio1.com/en/ (English version) and http://www.graphbio1.com/ (Chinese version). The source code of GraphBio is available at https://github.com/databio2022/GraphBio.

## Introduction

With the advance of high-throughput techniques (Goodwin et al., 2016), more and more researchers have started to depict molecular profiling in a systematic manner (Cancer Genome Atlas, 2012; Yan et al., 2015; Cancer Genome Atlas Research Network. Electronic address and Cancer Genome Atlas Research, 2017; Wang et al., 2018; Zhao et al., 2022). Massive amounts of omics data are produced and usually require sophisticated visualization analysis. For example, gene expression studies frequently use heatmaps, volcano plots, and MA plots to characterize expression changes from thousands of genes (Conesa et al., 2016). Moreover, principal component analysis (PCA) and correlation analysis are widely used to estimate similarity or dissimilarity between samples or groups. Although these methods are popular in omics research, they were usually published as R packages, such as ggplot2 (Wickham, 2016), pheatmap for drawing heatmap, GOplot for drawing chord plot of Gene Ontology (GO) analysis results (Walter et al., 2015), FactoMineR for performing PCA analysis (Lê et al., 2008), and pROC for drawing receiver operating characteristic (ROC) curves (Robin et al., 2011), which require experimental biologists to have good programming skills.

To address this problem, some bioinformatics scientists have recently developed some web-based tools to facilitate experimental biologists to conduct omics data visualization analysis easily, such as ggVolcanoR (Mullan et al., 2021), VolcaNoseR (Goedhart and Luijsterburg, 2020), ClustVis (Metsalu and Vilo, 2015), ImageGP (Chen et al., 2022), iGEAK (Choi and Ratner, 2019), and GENAVi (Reyes et al., 2019). However, these tools come with limitations, such as many unnecessary parameters, single function, input formats, and outdated color.

Herein, we developed an online web application called GraphBio using a shiny framework in R software. In comparison to other web tools, GraphBio specifically focuses on facilitating the generation of publication-ready plots easily and rapidly instead of data preprocessing and computing. Users can easily prepare data to be visualized by Excel software based on given reference example files from GraphBio. The default figures are generally ideal for publication, and they are only fine-tuned in some cases. We anticipate that GraphBio would become a good research tool for experimental biologists and advance new scientific discoveries.

## Materials and methods

GraphBio was built using R package bs4Dash (Granjon, 2022), a Bootstrap 4 style-based Shiny dashboard web application framework, in R Studio and R software (version 4.0.3). All plots generated by GraphBio are entirely based on popular R packages, including ggplot2 (Wickham, 2016), pheatmap, survminer, pROC (Robin et al., 2011), FactoMineR (Lê et al., 2008), factoextra, and GOplot (Walter et al., 2015). As an online web application, it is freely available at http://www.graphbio1.com/en/(English version) and http://www.graphbio1.com/(Chinese version) without login requirements. It can be accessed through any web browser, such as Google Chrome and Microsoft Edge. The source code of GraphBio is available at https://github.com/databio2022/GraphBio.

## Results and discussion

### Overview of GraphBio

**FIGURE 1:**
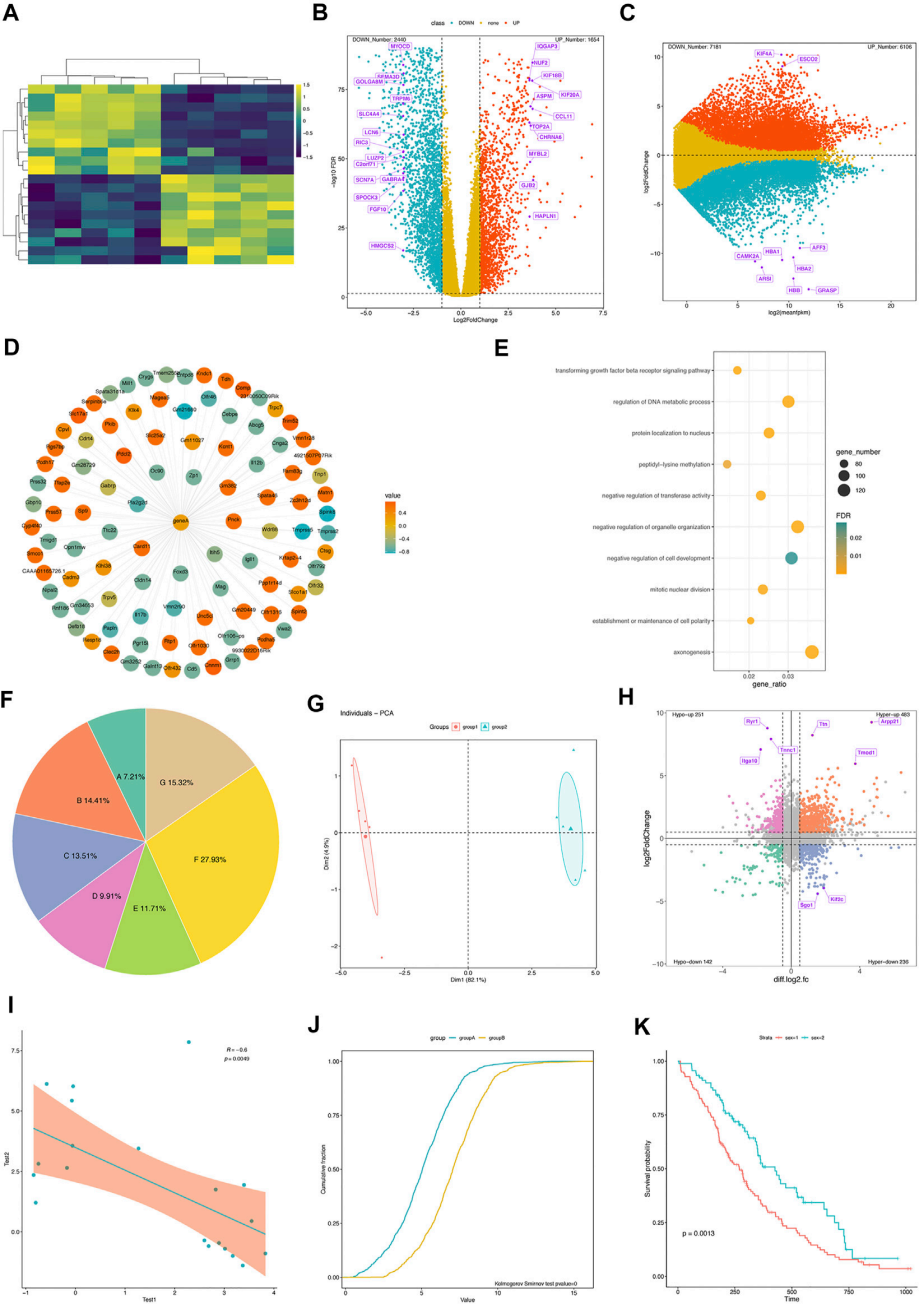
Flow diagram of the retrospective protocol for case inclusion and exclusion and data collection. Some representative visualization results from GraphBio. **(A)** Gene expression heatmap. Rows represent genes, and columns represent samples. Yellow represents upregulation, and blue represents downregulation. **(B)** The volcano plot shows significantly differentially expressed genes. The *x*-axis represents log_2_-transformed fold changes, and the *y*-axis represents −log_10_ (FDR). False discovery rate, FDR. Red represents upregulation, blue represents downregulation, and yellow represents genes that are not statistically significant. Some genes of interest are marked in purple. **(C)** MA plot shows significantly differentially expressed genes. The *x*-axis represents mean expression values of genes, and the *y*-axis represents log_2_-transformed fold changes. Red represents upregulation, blue represents downregulation, and yellow represents genes that are not statistically significant. Some genes of interest are marked in purple. **(D)** The network plot shows a group of expression-related genes for a target gene. The correlation values are calculated using the Pearson correlation analysis. Red represents positive correlation, and blue represents negative correlation. **(E)** The dot plot shows some biological processes of interest. The *x*-axis represents the gene ratio. The point size represents gene numbers. Color represents significance. **(F)** Pie plot. **(G)** PCA analysis. **(H)** The four-quadrant diagram shows the overlapped genes between RNA-seq and m6A-seq data. The *x*-axis represents log_2_-transformed fold changes of m6A-seq data, and the y-axis represents log_2_-transformed fold changes of RNA-seq data. Significant genes are marked in four different colors. **(I)** The Pearson correlation analysis between two variables. **(J)** Cumulative distribution curves. Kolmogorov–Smirnov test is used for comparing two samples. **(K)** Survival curves. Log-rank test is used for comparing two samples.

GraphBio provides 15 visualization analysis modules, including heatmap, volcano plots, MA plots, network plots, dot plots, chord plots, pie plots, four quadrant diagrams, Venn diagrams, cumulative distribution curves, PCA, survival analysis, ROC analysis, correlation analysis, and text cluster analysis. Some representative visualization results are shown in Figure 1. GraphBio supports four input file formats: csv, txt, xls, and xlsx. Notably, csv files cannot be encoded in UTF-8 and txt files must be tab-separated. All plots can be downloaded in a PDF, PNG, JPEG, or TIFF format with customizable size and resolution. A “run example” button was added to each module for users to learn the corresponding function features quickly without data preparation steps. Users can easily try changing default parameter values to observe the changes in the example figures. The example data are partly shown when users click “view example file” button to facilitate users to prepare their own data files. In addition, we have also added a “Help Center” section as a user manual in GraphBio.

Example 1 Heatmap for gene expression profiles.

Heatmap is a data matrix visualizing values in the cells using a color gradient, and it has been frequently used in omics data analysis. In GraphBio, the “Heatmap” module requires a gene expression matrix file as input. Our example data include 20 genes and 10 samples. A well-prepared csv file was uploaded, and then an idea plot was automatically generated (Figure 1A). We can easily adjust the plot by changing colors, clustering methods, gene, and sample name presentation as needed. Notably, the module provides six popular color presets, which are commonly presented in many papers, and a colorblind-friendly color was selected as default.

Example 2 Volcano plots for differential expression analysis.

Volcano plots depict the relationship between significance and fold changes of differentially expressed genes, and genes presented in the upper-left and upper-right corners are generally interesting to biologists. The “Volcano plot” module of GraphBio requires an input file with four columns, including geneID, log_2_ (fold change), significance (p or padj values), and label. The “label” column represents the genes to be highlighted on the figure. The example result is clearly shown in Figure 1B. The numbers of upregulated and downregulated genes were summarized. We can also customize the colors, fold changes, significance threshold, point size, and label size.

Example 3 Four quadrant diagrams for differential expression analysis between two omics data sets.

Four quadrant diagrams are generally used to analyze two omics data sets, such as RNA-seq and m6A-seq. We used differentially expressed genes and peaks from RNA-seq and m6A-seq data as a demonstrated example. The input file included six columns, including geneID, log_2_FoldChanges (RNA-seq), significance (p or padj values, RNA-seq), log_2_FoldChanges (m6A-seq), significance (p or padj values, m6A-seq), and label. The “label” column represents genes to be highlighted on the figure. The resulting figure is shown in Figure 1H. Four groups of genes are highlighted in different colors, and corresponding gene numbers are also summarized. We can also adjust the significance threshold, fold changes, point size, label size, and colors.

## Conclusion

In this article, we introduce GraphBio, an online web application for omics data visualization. It includes 15 popular visualization analysis methods, such as heatmaps, volcano plots, and MA plots. Experimental biologists can easily perform online analysis and get publication-ready plots *via* accessing the website http://www.graphbio1.com/en/(English version) or http://www.graphbio1.com/(Chinese version) using any web browsers like Google Chrome and Microsoft Edge. In the future, we will continue integrating more popular visualization analysis methods into GraphBio and provide more support to the research community.

## Data Availability

The original contributions presented in the study are publicly available. These data can be found at: https://github.com/databio2022/GraphBio.

## Abbreviations

FDR, false discovery rate; GO, Gene Ontology; ROC, receiver operating characteristic; PCA, principal component analysis.

## References

[B1] Cancer Genome Atlas N. (2012). Comprehensive molecular portraits of human breast tumours. Nature 490, 61–70. 10.1038/nature11412 PubMed Abstract | 10.1038/nature11412 | Google Scholar 23000897 PMC3465532

[B2] Cancer Genome Atlas Research Network. Electronic address and Cancer Genome Atlas Research (2017). Comprehensive and integrative genomic characterization of hepatocellular carcinoma. Cell. 169, 1327–1341.e23. e1323. 10.1016/j.cell.2017.05.046 PubMed Abstract | 10.1016/j.cell.2017.05.046 | Google Scholar 28622513 PMC5680778

[B3] Chen T. Liu Y. X. Huang L. (2022). ImageGP: An easy-to-use data visualization web server for scientific researchers. iMeta 1, e5. 10.1002/imt2.5 10.1002/imt2.5 | Google Scholar PMC10989750 38867732

[B4] Choi K. Ratner N. (2019). iGEAK: an interactive gene expression analysis kit for seamless workflow using the R/shiny platform. BMC Genomics 20, 177. 10.1186/s12864-019-5548-x PubMed Abstract | 10.1186/s12864-019-5548-x | Google Scholar 30841853 PMC6404331

[B5] Conesa A. Madrigal P. Tarazona S. Gomez-Cabrero D. Cervera A. Mcpherson A. (2016). Erratum to: A survey of best practices for RNA-seq data analysis. Genome Biol. 17, 181. 10.1186/s13059-016-1047-4 PubMed Abstract | 10.1186/s13059-016-1047-4 | Google Scholar 27565134 PMC5000515

[B6] Goedhart J. Luijsterburg M. S. (2020). VolcaNoseR is a web app for creating, exploring, labeling and sharing volcano plots. Sci. Rep. 10, 20560. 10.1038/s41598-020-76603-3 PubMed Abstract | 10.1038/s41598-020-76603-3 | Google Scholar 33239692 PMC7689420

[B7] Goodwin S. Mcpherson J. D. Mccombie W. R. (2016). Coming of age: Ten years of next-generation sequencing technologies. Nat. Rev. Genet. 17, 333–351. 10.1038/nrg.2016.49 PubMed Abstract | 10.1038/nrg.2016.49 | Google Scholar 27184599 PMC10373632

[B8] Granjon D. (2022). bs4Dash: A 'Bootstrap 4' Version of 'shinydashboard'. Available at: https://rinterface.github.io/bs4Dash/index.html . Google Scholar

[B9] Lê S. Josse J. Husson F. (2008). FactoMineR: An R package for multivariate analysis. J. Stat. Softw. 25, 1–18. 10.18637/jss.v025.i01 10.18637/jss.v025.i01 | Google Scholar

[B10] Metsalu T. Vilo J. (2015). ClustVis: A web tool for visualizing clustering of multivariate data using principal component analysis and heatmap. Nucleic Acids Res. 43, W566–W570. 10.1093/nar/gkv468 PubMed Abstract | 10.1093/nar/gkv468 | Google Scholar 25969447 PMC4489295

[B11] Mullan K. A. Bramberger L. M. Munday P. R. Goncalves G. Revote J. Mifsud N. A. (2021). ggVolcanoR: A Shiny app for customizable visualization of differential expression datasets. Comput. Struct. Biotechnol. J. 19, 5735–5740. 10.1016/j.csbj.2021.10.020 PubMed Abstract | 10.1016/j.csbj.2021.10.020 | Google Scholar 34745458 PMC8551465

[B12] Reyes A. L. P. Silva T. C. Coetzee S. G. Plummer J. T. Davis B. D. Chen S. (2019). GENAVi: A shiny web application for gene expression normalization, analysis and visualization. BMC Genomics 20, 745. 10.1186/s12864-019-6073-7 PubMed Abstract | 10.1186/s12864-019-6073-7 | Google Scholar 31619158 PMC6796420

[B13] Robin X. Turck N. Hainard A. Tiberti N. Lisacek F. Sanchez J.-C. (2011). pROC: an open-source package for R and S+ to analyze and compare ROC curves. BMC Bioinforma. 12, 77. 10.1186/1471-2105-12-77 PubMed Abstract | 10.1186/1471-2105-12-77 | Google Scholar PMC3068975 21414208

[B14] Walter W. Sánchez-Cabo F. Ricote M. J. B. (2015). GOplot: an R package for visually combining expression data with functional analysis. Bioinformatics 31, 2912–2914. 10.1093/bioinformatics/btv300 PubMed Abstract | 10.1093/bioinformatics/btv300 | Google Scholar 25964631

[B15] Wang Z. L. Li B. Luo Y. X. Lin Q. Liu S. R. Zhang X. Q. (2018). Comprehensive genomic characterization of RNA-binding proteins across human cancers. Cell. Rep. 22, 286–298. 10.1016/j.celrep.2017.12.035 PubMed Abstract | 10.1016/j.celrep.2017.12.035 | Google Scholar 29298429

[B16] Wickham H. (2016). Ggplot2: Elegant graphics for data analysis. New York: Springer-Verlag. Google Scholar

[B17] Yan X. Hu Z. Feng Y. Hu X. Yuan J. Zhao S. D. (2015). Comprehensive genomic characterization of long non-coding RNAs across human cancers. Cancer Cell. 28, 529–540. 10.1016/j.ccell.2015.09.006 PubMed Abstract | 10.1016/j.ccell.2015.09.006 | Google Scholar 26461095 PMC4777353

[B18] Zhao T. Tang X. Li D. Zhao J. Zhou R. Shu F. (2022). Prenatal exposure to environmentally relevant levels of PBDE-99 leads to testicular dysgenesis with steroidogenesis disorders. J. Hazard. Mat. 424, 127547. 10.1016/j.jhazmat.2021.127547 10.1016/j.jhazmat.2021.127547 | Google Scholar 34879533

